# On the relationship of perceived inequality and policy preferences

**DOI:** 10.1371/journal.pone.0341298

**Published:** 2026-08-19

**Authors:** Abraham Aldama, Cristina Bicchieri, Jana Freundt

**Affiliations:** 1 Vote Ref, Los Angeles, California, United States of America; 2 University of Pennsylvania, Center for Social Norms and Behavioral Dynamics, Philadelphia, Pennsylvania, United States of America; 3 Lucerne University of Applied Sciences and Arts, Walter-von-Moos-Promenade 1, Lucerne, Switzerland; Virginia Commonwealth University, UNITED STATES OF AMERICA

## Abstract

This paper investigates the determinants of individuals’ support for redistribution in the US from a behavioral perspective. In a comprehensive survey experiment with a large sample of US Americans, we identify participants’ (mis-)perceptions about income inequality together with their policy preferences and fairness views. US Americans underestimate the income earned by people at the bottom of the distribution and overestimate the income of top earners. There is no evidence for a causal effect of perceived inequality on policy views or political behavior. We find precisely estimated and robust null effects across different types of inequality beliefs and across different measures of support for redistribution. The null effect holds for different income groups and party affiliations, as well as for participants with different levels of trust in government and of perceived autonomy. Taken together, our results do not offer support for information about the extent of inequality being able to increase support for redistributive policies.

## 1 Introduction

Despite a sustained increase in income inequality in the US since the 1950s [1,2], taxes have not followed suit. Why has higher inequality not led to a higher demand for redistribution? Standard rational choice models, such as the general equilibrium model by [3], predict that higher income inequality produces a demand for more redistribution financed by taxes through the preferences of the median voter (whose relative income increases). Several studies have empirically addressed this question and have found mixed general results [4–13]. Thus, there is no clear empirical evidence for or against the positive association between inequality and demand for redistribution suggested by the canonical models. Reasons why the relationship might not be found likely include both supply and demand factors. Even if citizens demand greater redistribution, politicians may not be willing to respond and pass legislation in favor of higher taxation and redistribution. Increasing political polarization and the influence of corporations in politics through campaign contributions may be reasons why legislators do not pass more progressive legislation (for reviews of this literature, see [14] and [15]). In this article, we look at the *demand* side.

Demand for greater redistribution of income and wealth in the face of increasing inequality may be thwarted by a number of factors. Importantly, demand is expected to depend on the perception of inequality in the voter population. If people do not have an adequate understanding of the extent of inequality in their society, then the economic trend towards greater inequality may not translate into changes in voter’s preferences over social policies. This consideration is especially important since previous studies from different countries suggest that people might not always be well informed about the amount of inequality prevalent in their society [16–19]. Similar findings about other economic facts such as immigration support this idea. For example, US Americans overestimate the percentage of immigrants and minority groups [20–24]. In particular in the US, people seem to underestimate both income [17] and wealth inequality [18]—although this result has not been found in all studies (e.g., see [25]).

In order to argue that these beliefs are an important determinant of the demand for distribution, we have to show that they can indeed causally influence preferences. Previous surveys studied the relationship between economic facts and policy preferences (among others, [25–27]) and have found mixed results. Such mixed results can either point at a lack of a stable causal relationship or they can mask important effects among subgroups of the population. Moreover, the effects can possibly vary with how exactly beliefs about inequality and redistribution preferences are measured. Our experimental study is designed to cleanly identify a causal effect of inequality beliefs on policy preferences using between-subject treatments and to assess its robustness and generality. To account for the previous incoherent results, our survey design includes different measures for preferences over redistribution and systematically considers channels for this relationship that have been suggested in previous work in political science and economics.

Starting from the lack of coherent results in this literature, we investigate some of the reasons behind these findings. In particular, our study identifies channels through which this relationship might work—if a causal effect exists at all. We designed a comprehensive and well-powered survey experiment to test explanations using information treatments. We implemented the survey experiment with a large sample of the US population. Participants are asked to provide beliefs about the extent of income inequality in the US and to report their policy views. Policy views are measured (a) as beliefs about the fairness of the income distribution in the US and (b) as support for redistribution by public and private means (e.g., charities). We complement these attitudinal measures with an incentivized behavioral measure of the willingness to privately redistribute through a donation to a charity. Beliefs about the income distribution are elicited as estimations of the share of total income generated in the US that is received by the richest part of the population (the top 1% of income earners) and the share received by the relatively poor (the bottom 50% of income earners). To assess a causal influence of beliefs about income inequality on policy views, we inform randomly chosen subgroups of participants about the respective true income shares.

We bridge the literature in psychology and economics and explore biases in the perception of inequality by comparing treatments in which participants receive or do not receive a monetary incentive for a correct belief. Incentivizing beliefs has become an established standard in economics but not in psychology and other social sciences. Yet, there is only a very small literature that explicitly tests if biases regarding economic and political facts—–in our case income inequality—–may be reduced through monetary payments [e.g., 28].

We find that participants on average largely overestimate income inequality by overestimating income at the top of the distribution and underestimate income at the bottom of the distribution in the US. The shares of income of the top 1% and the bottom 50% are both inflated, the latter somewhat more. The observation that participants overestimate inequality at the upper part of the income distribution does not replicate earlier findings suggesting that Americans tend to underestimate income inequality in the US [17–19].

Perceptions of inequality significantly correlate with most measures of policy views in our study – confirming previous results in the literature (see [29] and [19,30] for wage inequality). We then, however, assess the causal effect of inequality perceptions on policy views using information and incentive treatments and find precisely estimated null effects, which are confirmed by Bayes Factors close to 0. All three interventions, providing information about the income share of the top 1 percent, providing information about the income share of the bottom 50 percent, and giving monetary incentives for accuracy, successfully alter the distributions of beliefs about inequality but there is no evidence of a causal effect of perceived inequality on policy views or behavior.

Our study is designed to provide a nuanced understanding of the relationship between perceptions of inequality and policy preferences. First, within subjects, we measure policy preferences not only as support for redistribution by the government, but also as support for private redistribution, for example by charities, by means of a self-reported and an incentivized behavioral measure. With this, we allow for the possibility that higher perceived inequality increases support for aiding the poor—but not necessarily by the government. This can occur if people exhibit low trust in the government’s capability to run adequate programs or have a taste for small government in general.

Two between-subject treatments allow to separately assess the impact of information about inequality due to disproportionately high income shares of the top income earners versus inequality due to disproportionately low income shares received by lower income earners. In public debates, inequality is often discussed in the context of excessive wealth and income of the top one percent, however, it is unclear if and how an awareness of this kind of inequality leads to more demand for redistribution. In fact, some moral philosophers argue that high incomes are acceptable if the least well-off in a society enjoy a decent standard of living, e.g., [31]. Asking about inequality at the top versus at the bottom of the income distribution, incentivizing answers versus not and eliciting support for private and public redistribution does not substantially alter the conclusions we can draw from the data and it does not explain mixed findings for a causal effect on policy views.

Going beyond average treatment effects, we explore individual-level heterogeneity in the sample and analyze if treatment effects differ depending on one’s own economic situation as well as on some fundamental attitudes and preferences. Importantly, a lack of trust in government makes information about the income distribution less relevant for demanding redistributive policies, since then the government is not expected to effectively provide welfare programs that improve the economic situation of their citizens (see also the discussion in [32]).

In addition, perceived autonomy and perceived equality of opportunities have been shown to make people more accepting of inequality [25,33–36] and therefore have the potential to be important moderators of a causal effect on support for redistribution. Last but not least, we include political ideology, as expressed by Americans’ partisanship, as well as a person’s own income as potential moderators since they are closely related to both, the perception of inequality (e.g., [37]) and support for redistribution [38].

While most of these characteristics and attitudes are indeed correlated with how people perceive inequality and with political preferences in our data, *none* of them moderates a causal effect of the information and incentive treatments. The finding of a null effect holds for *all* subgroups we analyze. In fact, a handful of isolated significant results appear in the analysis, however, when controlling for multiple hypotheses testing, they disappear. This emphasizes the benefits of a more comprehensive investigation, that puts isolated statistically significant results into perspective instead of overinterpreting single effects (found by chance). The overall picture that emerges from our comprehensive approach is that there is no evidence of a causal relationship.

Previous literature has shown that people tend to misperceive inequality [16; 18, e.g.,], but previous results on the relationship between beliefs about inequality and policy views are inconclusive [25; 32, e.g.,]. Different from our approach, a number of studies have examined whether beliefs about *one’s own position* in the country’s income distribution may affect preferences for redistribution [32,39–42]. They find that providing this kind of information has limited effects on people’s demand for redistribution. If they find an effect, it tends to be in the direction of being self-serving: When people find out they are relatively poorer than they thought they were, they demand more redistribution and vice versa. Similarly, [43] and [44] found that being exposed to physical or visual reminders of inequality (e.g., a luxury car in a poor neighbourhood) leads to a shift in support for redistribution in a self-serving direction. Such self-serving motives are excluded in our study that investigates the role of beliefs about society. A few papers study the role of beliefs about a person’s socio-economic environment for preference formation in different political contexts such as social mobility, discrimination and immigration ([25]: beliefs about intergenerational mobility and preferences for redistribution, [45]: beliefs about racial labor market discrimination and support for affirmative action programs / donations to a civil rights organisation, [27]: beliefs about the gender wage gap and support for gender policies, [26]: beliefs about the share of the foreign born population and attitudes toward immigration policies). Our null-result for the role of beliefs about economic facts in shaping policy preferences is in line with evidence found in related domains such as immigration [26] and discrimination [27,45]. We speculate that such preferences are rather influenced by, for example, political ideology and life experiences and are possibly deeply rooted in a person’s social identity [46]. If the results from the information experiments hold true more generally, this may suggest that information interventions alone may not be an effective tool to increase voters’ support for policies, such as, taxation and welfare assistance.

## 2 Study design

### 2.1 Implementation

The survey was implemented on Prolific Academic (www.prolific.co) during the fall of 2020. It was programmed using the survey software Qualtrics [47]. We conducted the experiment between October 5 and November 10, 2020, including a soft launch on Oct 5 and a second soft launch on Oct 16. Then we collected data continuously from the 19th to the 23rd. Please note that 20 percent of the sample was not usable due to a programming mistake that did not affect randomization into treatment. In order to reach the pre-registered number of observations we collected 577 additional observations between November 6 and 10. Importantly, on November 3rd, a polarizing presidential election took place. We therefore conduct robustness checks to see if observations close to the election date produced different results.

We pre-registered the study at the Open Science Foundation, [48] provides the pre-registration, the full questionnaire as well as the dataset and code. Written ethics approval was obtained by the Institutional Review Board of the University of Pennsylvania on June 24, 2020 (case number 843423). Participants received information about data protection and provided informed consent on a first screen before entering the survey. Following [49], in order to have enough power to detect an effect of 0.15 standard deviations, which is common in information experiments, we sought to have at least 700 respondents in each treatment and therefore recruited a sample of 2,882 adults living in the US. We used age quotas to ensure our sample matched the distribution of ages in the US population.

A summary of characteristics of the sample and the US population can be found in S1 Table. Compared to the general adult population in the US, there are more lower income earners, more non-Hispanic whites and slightly more women (54.37%) in our sample. The median duration of the survey was about 12 minutes and all subjects were paid a base payment of $1.50 for completing it. Note that participants in one treatment were able to receive an incentive of $1 and every participant had a chance to be one of 10 out of 2,882 participants to be selected for a payment of $20 out of which they could donate a chosen amount to a charity, leading to an overall average payment of $1.58. Assignment to treatments was random. In order to ensure high quality responses, multiple attention checks were introduced throughout the survey. Subjects who failed three attention checks were excluded from further participation and from any payments. In addition, we included a short training question that allowed participants to get familiar with how to use the sliders in the survey. Before entering the study, every participant had to agree to a consent form informing them about the nature of the study and about their rights as a participant and they completed a reCaptcha.

### 2.2 Experimental design

**Fig 1:**
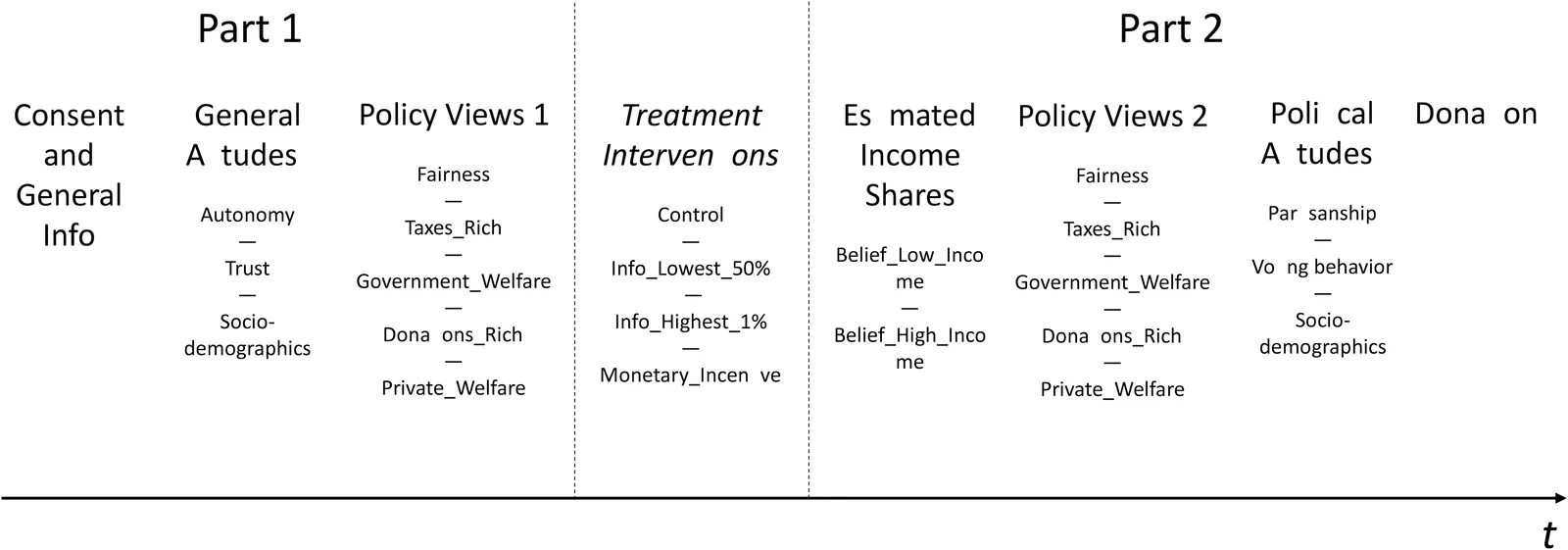
Timeline of the survey.

Fig 1 displays the timeline of the survey, the full questionnaire is linked in the supporting information. The survey consists of two parts, separated by a treatment intervention. We employ a between-subject treatment design. In part 1, participants were asked to give informed concent, then they were asked a few general attitude questions. Subsequently, they saw a question block on their policy views, followed by the respective treatment intervention. Each treatment intervention is accompanied by questions on beliefs about income shares that are asked directly after the treatment interventions. Afterwards, participants saw the block of questions on policy views once again. At the end of part 2, we added a second block with more questions on general attitudes, political attitudes and socio-demographics. Within each block, the order of questions was randomized. Before leaving the survey, everyone was given the chance to donate to a charity. In the following, each question block will be described in more detail.

**Policy Views.** Respondents answered five questions regarding fairness of the income distribution and preferences for redistribution through public as well as private means. The questions measure two different dimensions of social policy, namely “redistribution from” and “redistribution to” [50] by focusing either on taxing the rich or on providing welfare assistance to the poor. These five questions are our main outcome variables and they appear before and after the treatment interventions.

*Fairness: “How fair do you believe the income distribution in the United States is?”*

*Taxes_Rich: “Think about Federal income taxes. Do you think that income taxes for the richest 1% should be lower than they are now, stay the same, or higher than they are now?”*

*Government_Welfare: “Now think about government spending on aid to the poor such as food stamps and housing subsidies. Do you think it should be less than it is now, stay the same, or more than it is now?”*

*Donations_Rich: “Think about contributions to charities by US Americans. Do you think that the richest 1% should donate less, about the same amount, or more than they donate now?”*

*Private_Welfare: “Private organizations such as religious organizations and charities provide welfare assistance to vulnerable people such as children, the elderly, the disabled, and the very poor. Should these groups be spending more or less on welfare assistance?”*

**Fig 2:**
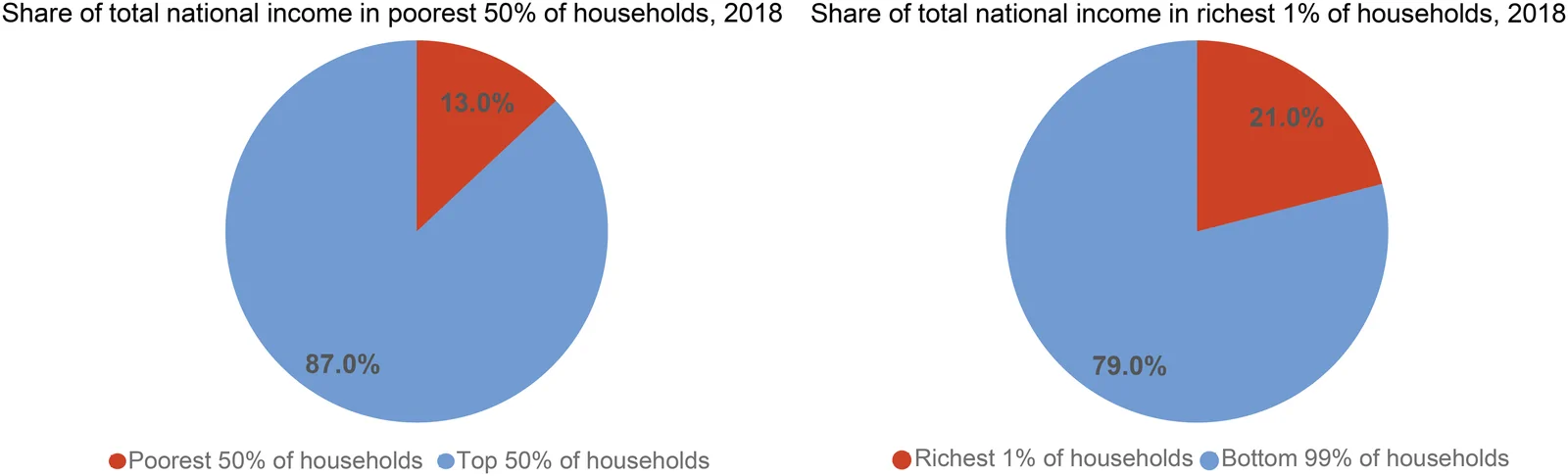
Information treatments received by subjects. A: Information in Info_Lowest_50%. B: Information in Info_Highest_1%.

**Treatments and Estimation of Income Shares.** After answering the five policy questions, respondents were randomly assigned to one of four treatment conditions. An overview of all treatments is provided in Table 1. In treatment *Control*, respondents did not receive any information, they were simply asked to provide their beliefs about income shares. In *Info_Lowest_50%*, they were shown information about the share of total income earned by the bottom 50 percent of the distribution in 2018 (the true share is 13%). In *Info_Highest_1%*, respondents were shown the percentage of income generated in 2018 received by the richest 1 percent of the distribution (the true share is 21%). The illustration we used to provide information to participants is displayed in Fig 2.

**Table 1:** Overview of treatments.

Treatment	Description	N
Control	No intervention	717
Info_Lowest_50%	Information on income share of bottom 50%	720
Info_Highest_1%	Information on income share of top 1%	722
Monetary_Incentive	Bonus for correct answers	723

In both information treatments, we showed them two pie charts displaying the share of income earners (50% and 1%, respectively) and the share of income they received (13% and 21%, respectively), allowing subjects to get an intuition about the proportions. Similar pie charts were used to elicit inequality beliefs. Each participant was asked to state which share of the total income generated in the US in 2018 was received by the bottom 50% and by the top 1% of the population in two separate questions. Participants were able to set the size of the slice of the pie while seeing the actual number in percent below. Finally, in *Monetary_Incentive*, we gave no information but told respondents that we pay them a bonus for correct answers. For each answer that was within five percentage points of the true percentage of income obtained by the top 1% and bottom 50% they were paid an additional $1.

**Additional Survey Questions.** In two blocks of questions, one at the beginning and one towards the end of the survey, participants were asked about their general and political attitudes plus some standard socio-demographic questions. These include questions regarding partisan identification, trust in various organizations, a battery of questions adapted from the Index of Autonomous Functioning [51], a question about how much control they believe other people have over their lives and their perception of equality of opportunity in the US.

**Donation.** Before exiting the survey, respondents were informed that we randomly select ten out of all survey participants to receive an additional payment of $20. They were also told that they can choose to donate any amount between 0$ and $20 to a charity and that the payment will become effective if they were selected to receive the 20$ payment.The respondents then selected one out of a list of five charities to receive the donation: The Salvation Army, Meals on Wheels, Scholarship America, Save the Children USA, and Equal Justice Initiative. This donation decision is our behavioral measure of subjects’ willingness to redistribute through private means.

### 2.3 Hypotheses

The main goal of our survey experiment is to determine whether and how perceived income inequality causally affects people’s policy preferences. We first assess participants’ beliefs about the income shares received by the bottom 50% and the top 1%, respectively, and test if average beliefs are successfully shifted by our treatment interventions compared to *Control*. We refrain from stating any hypotheses about the direction of the changes in beliefs about the income distribution.

**Hypothesis 1** (Info_Lowest_50%). *Learning that the income share received by the lower income earners is smaller (larger) than their prior belief causes (1) an increase (decrease) in the demand for taxation of the rich (Taxes_Rich) and for charitable donations (Donations_Rich) and (2) an increase (decrease) in support for private and public welfare spending (Government_Welfare, Private_Welfare). The effect on Fairness views is ambiguous.*
**Hypothesis 2** (Info_Highest_1%). *Learning that the income share received by the top income earners is larger (smaller) than their prior belief causes an increase (decrease) in demand for taxation of the rich (Taxes_Rich) and private charitable giving (Donations_Rich). We expect no effect on aid to the poor (Government_Welfare, Private_Welfare). The effect on Fairness views is again ambiguous.*

While beliefs about the (relative) income of the bottom income earners intuitively relate to support for aid to the poor, a perception of high inequality due to the (relative) affluence of high income earners, however, is not necessarily directly linked to support for aid to the poor. An indirect link can exist since people who hold this belief might find higher taxation acceptable or might expect the rich to voluntarily donate more, simply because they have more. This is why we believe that it’s crucial to allow for this distinction when testing the causal effect of inequality beliefs on support for redistributive policies. One can argue that the effect on support for aid to the poor will ultimately depend on if and how much people update their beliefs about how much income is earned by the poor after seeing *Info_Highest_1%*. This effect is more indirect and, especially, less salient, but the intervention will also mechanically lead to an update in people’s belief about the income received by the poor and with this it can influence their support for aid. We refrain from integrating this aspect in our main predictions since it is not salient in the intervention and might therefore not have a noticeable impact. We will, however, take it into account when analyzing the results.

Note that also the *moral reasoning* behind an increased demand for redistribution differs in the two cases: The first hypothesis is based on a need-based argument, i.e., higher (lower) poverty makes more (less) welfare efforts necessary in order to ensure a decent standard of living for everyone. In the second case, informing people that top income earners earn a larger (smaller) fraction of the total income than they previously thought, simply informs them that there is “a lot of money to be given away” while there is not necessarily a moral argument involved. The effect on fairness perceptions is unclear in both cases and we refrain from stating an ex ante hypothesis about the average effect. While we may on average expect lower fairness judgments when beliefs about poverty increase, individuals will hold different fairness views so that it is not clear what to expect on average without knowing the fairness ideals of the population. This aspect is discussed in more detail in the last paragraph of this section.

**Hypothesis 3** (Monetary_Incentive). *The belief that inequality is higher (lower) increases (decreases) support for taxation and private charitable giving (Taxes_Rich, Donations_Rich) as well as support for private and public aid to the poor (Government_Welfare, Private_Welfare). The effect on Fairness views is again ambiguous.*

Based on previous studies [28, e.g.,], we expect that monetary incentives attenuate people’s biases in beliefs about the income distribution and thus significantly alter perceptions of income inequality. The consequential shifts in policy views will follow the same mechanics as outlined above. The reason for updating one’s beliefs can be twofold: Monetary incentives can reduce errors in statistical reasoning because they make people think harder about the question. Second, the possibility to earn additional money can make people more likely to report their true beliefs, i.e., it may reduce their ideological bias if they are (at least partly) aware of it. Note, however, that some subconscious biases might survive and influence a person’s beliefs even if she thinks hard about the problem. If a person is not aware of her bias, she might intend to best answer the question, but still not be able to overcome a deeply rooted subconscious ideological (or statistical) bias.

**Hypothesis 4** (Moderators). *We expect treatment effects to be moderated by own income, partisanship, trust in government and perceived autonomy and equality of opportunity: When people update their beliefs towards believing there is more inequality, the effects on support for redistributive policies should be stronger for people with lower household income, for Democrats, for people with lower perceived autonomy and equality of opportunity and for people with higher levels of trust in government.*

Whether or not—and how strongly—the relationships outlined in the main hypotheses exist is expected to depend on own household income, partisanship, trust in government, own perceived autonomy as well as on perceptions of others’ autonomy and equality of opportunity. Average treatment effects can hide differential effects on subgroups and we expect to find heterogeneity in effects on policy views for the following factors:

*Own Income:* A relatively rich person, who would be negatively affected by redistribution, might not necessarily support more redistribution when being reminded about the prevalence of inequality because it involves a personal cost (see also [41,43]). Conversely, we might expect the poor, who would benefit from redistribution, to support more redistribution once they learn that the rich receive an even higher income share than they thought [42,44].

*Partisanship:* Democrats and Republicans have different views on taxation and social welfare [52, e.g.,]. Moreover, any information about an ideologically loaded topic such as inequality may lead partisans to become more polarized [38]. According to [38], as a result of increased political polarization, liberal voters who are more politically informed tend to be more supportive of redistributive policies than those with low information while more informed conservative voters tend to be less supportive of redistributive policies than those with low information. We thus hypothesize that providing information about the extent of inequality in the US will have different effects depending on a participants’ political partisanship. Previous studies show heterogeneous results with respect to this prediction. [25] find that preferences for redistribution are related to perceptions of social mobility only among left-wing respondents. In contrast, [45] find an overestimation of racial discrimination by Democrats, but no explanatory power of partisanship for the relationship to political behavior.

*Trust:* Previous studies suggest that people’s trust in government is strongly and positively correlated with their support for redistribution by public means [32,53,54]. Note that, however, there are also studies that disagree: for instance [55] argues that increasing trust may not influence preferences for redistribution. Especially for the US, previous studies have found relatively low levels of trust in government. [32], among others, have suggested that trust might play a role in explaining why high perceived inequality might not translate into support for more government welfare spending. Knowledge about inequality should only translate into support for more redistribution by the government if a person trusts that the government will do a good job in efficiently collecting and redistributing money and providing public goods. Independent of one’s trust in the abilities of the government one might, however, still be in favor of helping the poor, thus supporting redistribution by *private* means such as charitable giving. Thus, while participants with low trust are expected to show no treatment effect on support for public redistribution, we expect to find an effect on support for redistribution through private means.

*Autonomy and equal chances:* Holding a stronger belief in personal autonomy is associated with a greater role of personal responsibility for one’s own life outcomes. Our own previous work as well as other studies suggest that people’s sense of autonomy, or the amount of control they believe they have over their own lives, affects their judgement of the fairness of existing inequality, as well as their belief *how much* inequality exists [37]. It has also been shown that just priming the idea of choice makes people more comfortable with inequality [56,57]. Relatedly, a stronger belief in equality of opportunity means that a person believes that everyone faces relatively similar starting conditions. Both beliefs are important ingredients for believing in a just meritocratic society. Meritocrats judge income inequalities to be fair if everyone had the chance to be successful, hence people are responsible for their situation and differences in income rather reflect differences in effort and hard work. Several studies have shown that meritocratic fairness views are indeed prevalent in the US and elsewhere, see for example [36]. People with a meritocratic view will not necessarily see a higher need for welfare interventions when they learn that income inequality in the US is higher than they previously thought. A series of studies have established a strong link between beliefs in equality of opportunity and preferences for redistribution [36,58,59]. Generally speaking, given any income distribution, people will only wish to redistribute from high to low income earners if they consider the allocation of income to be *unfair*. Previous experimental and empirical studies have shown that people accept much higher income inequality and demand less redistribution if they believe that everyone had the same chances to be successful [35,36] and that upward economic mobility exists [25,33,34]. We expect the effects of higher perceived inequality on preferences for (public and private) redistribution to be smaller for people with high perceived own and others’ autonomy and with strong beliefs in equality of opportunity. Note that the same line of reasoning leads us to refrain from stating a hypothesis about an effect of each intervention on the variable Fairness. The average effect will strongly depend on the fraction of meritocrats in the sample.

## 3 Results

**Fig 3:**
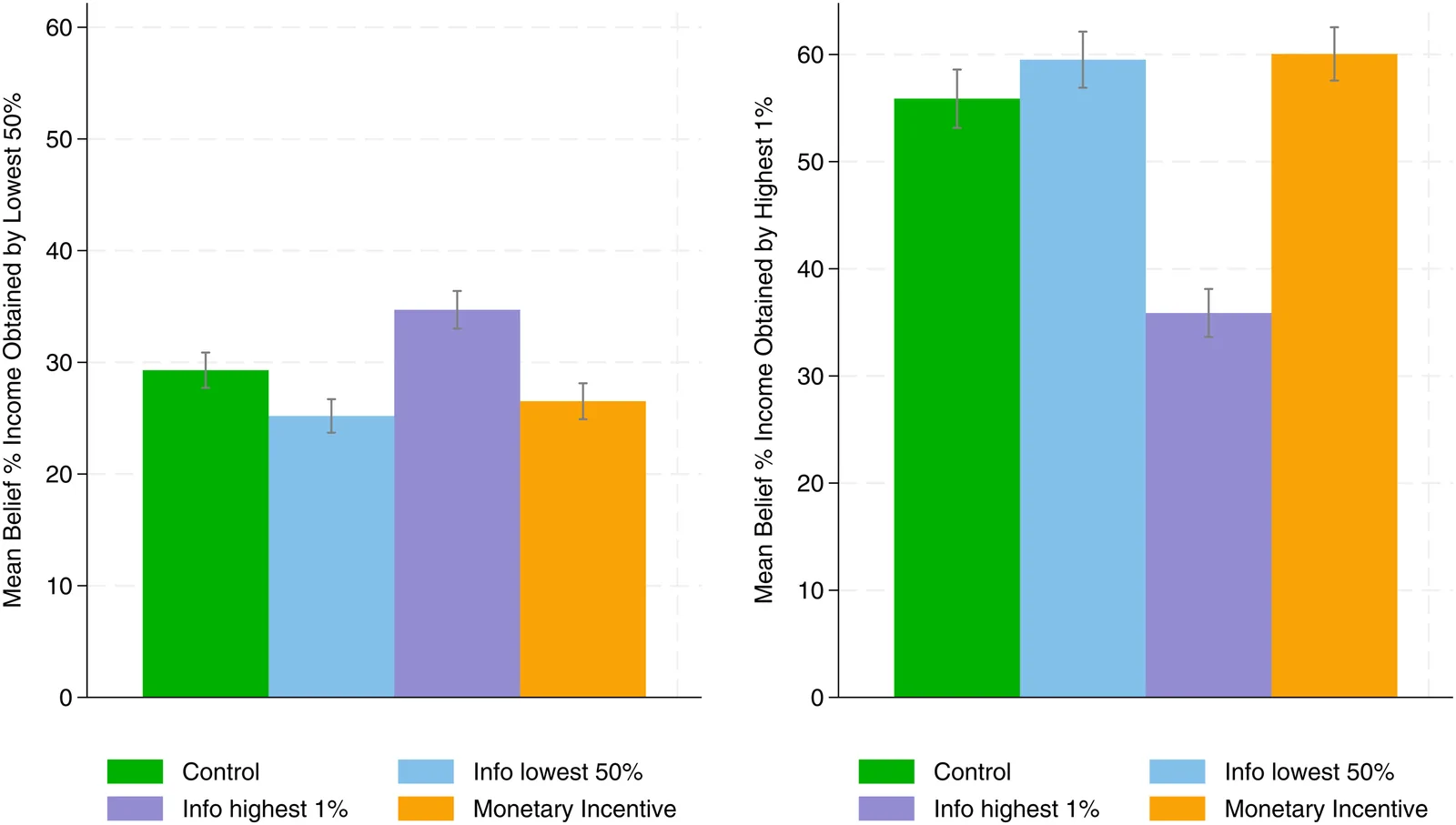
Average beliefs about the share of total income received by each income group (with 95 percent CI). A: Beliefs about income share received by the highest 1% of income earners. B: Beliefs about income share received by the lowest 50% of income earners.

**Misperceptions of inequality.** We begin by reporting average beliefs about the distribution of total income in the absence of a treatment intervention. In *Control*, on average respondents state they believe that the top 1% received 55.9 percent and that the bottom 50% received 29.3 percent of the total income generated in the US in 2018, see Table 2 and Fig 3. The true numbers are 21 percent and 13 percent, respectively. The percentage of respondents in the *Control* treatment who report the income shares accurately, meaning within 5 percentage points of the true value, is 23.9% for the bottom 50% and 2.5% for the top 1%. This indicates a rather low accuracy in beliefs, especially with regard to the income share of the top 1% of income earners.

**Table 2:** Stated beliefs about the share of total income received by the lowest 50% and by the highest 1% of income earners in percent, by treatment.

	Belief Lowest 50%	Belief Highest 1%
Control	29.3	55.9
	(21.6)	(37.1)
Info Lowest 50%	25.2	59.5
	(20.6)	(35.8)
Info Highest 1%	34.7	35.9
	(23.1)	(30.7)
Monetary Incentive	26.5	60.0
	(22.1)	(34.1)
True Values	13	21

Standard deviations in parentheses.

While respondents correctly believe the top (bottom) income earners to earn a larger (smaller) income share compared to their share of the total population, their beliefs about income shares are somewhat inflated: Beliefs about the share of income going to the bottom 50% of the distribution are about 16 percentage points higher (225.4 percent of the true value) and beliefs about the share of the top 1% are 35 percentage points higher (266.2 percent of the true value). Thus, both income shares are largely overestimated. Beliefs about the income share of the top earners are more inflated and less accurate.

**Result 1.** *Respondents on average largely overestimate income inequality at the top of the distribution and underestimate income inequality at the bottom of the distribution in the US. Beliefs about income shares of the bottom 50% and the top 1% are both largely inflated, the latter somewhat more (29.3 compared to 13 percent and 55.9 compared to 21 percent).*

**Treatment interventions and beliefs.** Fig 3 and Table 2 indicate that beliefs about both income shares have been successfully altered by our treatment interventions. Beliefs about the income share received by the top 1% are overall high and even slightly higher in *Monetary_Incentive* and *Info_Lowest_50%* than in *Control* (60.0 percent and 59.5 percent compared to 55.9 percent (both statistically significant, *p* = 0.03 with d.f. = 1,438 and *p* = 0.06 with d.f. = 1435, two-tailed t-tests)). Compared with these beliefs, we observe that the information about the true value (21 percent) in *Info_Highest_1%* successfully and drastically shifted average beliefs to 35.9 percent (20 percentage points lower than in *Control*, *p* < 0.01, d.f. = 1,437, two-tailed t-test).

Giving subjects information about the true value (13 percent) as well as incentivizing them to think harder about the question both lead to a significant update in the right direction (25.2 percent (*p* < 0.01 with d.f. = 1,435, two-tailed t-test) in *Info_Lowest_50%* and 26.5 percent *Monetary_Incentive* (*p* = 0.02 with d.f. = 1,438, two-tailed t-test). Beliefs are mechanically pulled up in *Info_Highest_1%* after receiving the information that the top earners receive less than they thought (34.7 percent compared to 29.3 percent in *Control* (*p* < 0.01 with d.f. = 1,437, two-tailed t-test). The accuracy of beliefs, defined as being within 5 percentage points of the true value, increases strongly compared to *Control* (*Info_Lowest_50%*: 52.2% for the bottom 50% and 4.6% for the top 1%; *Info_Highest_1%*: 17.9% and 44.5%; *Monetary_Incentive*: 27.7% and 6.6%), albeit there is still a substantial fraction of respondents who report inaccurate beliefs. Overall, stated beliefs are largely inflated and both information interventions successfully shift respondents’ beliefs about the income distribution. However, they also demonstrate that a non-negligible fraction of individuals seem to have ignored the information and do not (perfectly) update their reported beliefs. These participants may have been inattentive or may have imperfect memory, however, we cannot empirically distinguish between reasons for this updating behavior.

It is particularly noticeable that receiving information about the top 1% of the distribution strongly reduces participant’s average beliefs to state that the top 1% take home a 20 percentage points smaller proportion of the total income (and that the bottom 50% take home a 5.4 percentage points higher proportion). Thus, the intervention in *Info_Highest_1%* leads people to on average believe there is *less* inequality. On the other hand, both *Info_Lowest_50%* and *Monetary_Incentive* lead respondents to state that the top 1% take home a higher share and that the bottom 50% take home a smaller share compared to beliefs in the control treatment. This means that, on average, these two treatments lead subjects to believe that there is *more* inequality. The first observation goes against the results of some previous studies that suggested that US Americans tend to underestimate inequality and thus information interventions should lead to a higher (and more precise) belief about inequality. With this, our results offer an interesting possibility to test how changes in the perception of inequality in both directions, upward and downward, causally impact policy views and preferences.

**Causal effects on policy views.** Table 3 shows the difference-in-difference estimates of our main outcome variables for all three treatments. We see that, in general, before treatment there are no statistically significant and no meaningful differences across treatment groups in how respondents answer the policy questions. The only exception is a minor higher average response to the question about taxing the rich (*Taxes_Rich*) in *Info_Lowest_50%*. The *Post-Treatment* variable in Table 3 shows how answers to all outcome variables change in *Control*, i.e., without an intervention. We observe a minor drift towards stating the income distribution in the US is less fair and that the government should spend more on welfare. Overall, participants in the control condition provide answers consistent with their first answer when they are asked the questions for the second time. The interaction terms in Table 3 then show to what extent changes in policy views in part 2 are different from the ones in *Control* in each of the three treatment conditions. Across treatments, we see that the interventions have a limited impact on changes in participants’ beliefs about the fairness of the income distribution and in their preferences for redistribution. Only *Info_Highest_1%* has a statistically significant effect (compared to *Control*) in increasing people’s support for more government aid for the poor (*Government_Welfare*), albeit very small in magnitude (1.2 points on a 100-point scale). Furthermore, as shown in S4 Table, this effect disappears when using sharpened q-values to correct for false discovery rates when testing multiple hypotheses [60,61].

**Table 3:** Treatments on changes in Fairness, Taxes_Rich, Government_Welfare, Donations_Rich and Private_Welfare between part 1 and 2, OLS with robust standard errors.

	Fairness	Taxes	Government	Donations	Private
		Rich	Welfare	Rich	Welfare
Info_Lowest_50%	−2.007	2.692**	1.929	.693	.674
	(1.554)	(1.118)	(1.240)	(1.021)	(1.014)
Info_Highest_1%	−1.793	1.521	.854	1.737*	1.515
	(1.560)	(1.163)	(1.266)	(1.000)	(1.027)
Monetary_Incentive	.037	1.073	.469	.246	−.009
	(1.570)	(1.146)	(1.294)	(1.022)	(1.068)
Post-Treatment	−1.855***	.662	1.107***	.351	.141
	(.506)	(.456)	(.369)	(.479)	(.442)
Info_Lowest_50% × Post-Treatment	−1.227	−.222	.126	.519	.612
	(.746)	(.574)	(.542)	(.678)	(.598)
Info_Highest_1% × Post-Treatment	−.824	.109	1.207**	−.496	.934
	(.701)	(.600)	(.537)	(.660)	(.634)
Monetary_Incentive × Post-Treatment	−1.029	−.307	.294	−.032	1.058
	(.713)	(.632)	(.600)	(.673)	(.672)
Constant	31.767***	80.038***	70.621***	79.026***	70.469***
	(1.112)	(.829)	(.914)	(.718)	(.743)
N	5,764	5,764	5,764	5,764	5,764
Bayes Factor (Info_Lowest_50%)	.004	.026	.002	.000	.000
Bayes Factor (Info_Highest_1%)	.002	.001	.001	.001	.005
Bayes Factor (Monetary_Incentive)	.000	.000	.000	.000	.000

Robust standard errors clustered at the individual level in parentheses.

^*^p < 0.1, ^**^ *p* < 0.05, ^***^ *p* < 0.01.

Recall that *Info_Lowest_50%* and *Monetary_Incentive* make people perceive more inequality while *Info_Highest_1%* makes participants perceive less inequality. If the treatments had any impact on people’s preferences for redistribution, we would expect the effects of *Info_Lowest_50%* and *Monetary_Incentive* go in the same direction, opposite to that of *Info_Highest_1%*. The results show no such pattern. Rather, the results show consistently that the treatments do not significantly affect preferences for redistribution through either public or private means.

Baseline is Control. For each variable, the reported Bayes Factors compare models including the respective treatment with models including only the post-treatment variable.

**Robustness checks.** In this section we address a variety of concerns about what might be influencing the results. Note that since we use a diff-in-diff approach, adding demographic covariates as control variables to our regressions does not help to increase the efficiency of our estimates since individual fixed effects are already controlled for. One concern may be that there are effects but that they are particularly ephemeral. As respondents answer a series of questions, treatment effects might wash out by the time they are responding if a question appears later in the survey. In S5 Table we use the fact that the order of questions within each block is randomized and repeat our estimations with only those participants who saw the relevant question first within the block on policy views in part 2, see Fig 1. Our finding that each treatment condition does not statistically significantly affect perceptions of fairness or support for public or private redistribution holds when we analyze only the responses of participants who randomly answered the question immediately after receiving the treatment. Finally, another concern is that the lack of results may be due to respondents not paying attention to the survey. As noted in Section 2.1, we had several attention checks throughout the survey. Respondents failing three attention checks were terminated and not allowed to complete the survey. In S6 Table we restrict our analyses to participants who never failed a single attention check and can thus be classified as “attentive”. Again, the results are consistent with the findings in our main analysis. Another concern that may arise is that only people who actually “take up the treatment” might be changing their preferences about redistribution. To address this concern, we run an instrumental variables regression for each treatment and outcome in which we code participants as receiving the treatment if their response is within five percentage points of the true corresponding quantity, i.e., participants in Info_Lowest_50% are coded as receiving the treatment if their response is between 8 and 18 percent; similarly participants in Info_Highest_1% are coded as receiving the treatment if their response is between 16 and 26 percent. For the treatment Monetary_Incentive, we run the analysis twice, coding treatment received for each of the two income shares. The results of these estimations confirm our prior result of no changes in preferences for redistribution. The only significant result disappears once we correct for multiple hypothesis testing. See S7 to S10 Tables for the full results. Finally, to address concerns that there might be differences between respondents answering before versus after the presidential election on November 3rd, 2020, we interact the treatment with an indicator for having answered the survey in November. The results in S18 Table show that there are no meaningful patterns around the election.

Taken together, we observe that, even though all treatments impact people’s beliefs about the extent of inequality in the US, they do not have a statistically significant impact on fairness views nor on the demand for redistribution through either public or private means.

In line with this result, the treatments had no statistically significant effect, on average, on respondents’ donations to charity (Table 4). None of the treatments affected either the amount donated to charity (column (1)) or the decision whether or not to make a donation (column (2)). Bayes factors for all specifications in Tables 3 and 4 are close to zero and with this confirm our results.

**Table 4:** Treatments on amount donated in USD (out of 20 USD, *Amount_Donated*) and on the binary decision whether or not to donate, *Donation_Decisions* = 1 if *Amount_Donated*>0, 0 otherwise.

	Amount_Donated	Donation_Decision
Info_Lowest_50%	.249	.015
	(.346)	(.023)
Info_Highest_1%	.459	.021
	(.348)	(.023)
Monetary_Incentive	.060	−.008
	(.533)	(.023)
Constant	7.175***	.749***
	(.247)	(.016)
N	2,882	2,882
Bayes Factor (Info_Lowest_50%)	.004	.000
Bayes Factor (Info_Highest_1%)	.008	.003
Bayes Factor (Monetary_Incentive)	.006	.000

Robust standard errors in parentheses.

^*^p < 0.1, ^**^ *p* < 0.05, ^***^ *p* < 0.01.

OLS with robust standard errors, Baseline is Control. For each variable the reported Bayes Factor compares models including the respective treatment condition to a model only including a constant.

**Result 2.** *We find no evidence for a causal effect of beliefs about inequality on policy preferences. Giving people information about the income distribution does not have a statistically significant effect on average fairness views, support for redistribution by the government or by private organizations, or the willingness to donate to the poor. Hypotheses 1, 2 and 3 are thus not supported by our evidence.*

This result may be surprising given that both beliefs about income shares are significantly correlated to several of the covariates, especially to *Fairness* and *Taxes_Rich*, see Table??. This underscores the importance of identifying causal relationships since the correlation between beliefs about inequality and policy preferences my very likely suffer from endogeneity or omitted variables.

Does this lack of a statistically significant result in average treatment effects hide changes in policy views of subgroups that cancel out when aggregating all responses? The lack of changes in average views could be driven by differential changes in a heterogeneous population if certain subgroups of participants respond differently than others to the treatments. We therefore now explore possible moderators of treatment effects.

**Heterogeneous treatment effects.** We test the following moderator variables: own income, partisanship, trust in government, perception of own and others’ autonomy, and the belief in equality of opportunity. *Income* is measured in five categories, which roughly correspond to the household income quintiles in the US in 2018: less than $25,000, between $25,000 and $50,000, between $50,000 and $80,000, between $80,000 and $130,000, and more than $130,000. *Partisanship* is measured on a 7-point scale, using the same questions as the American National Election Survey to code people from Strong Democrats (1) to Strong Republicans (7). Participants are asked to self-identify as Democrat, Republican or Independent and then to state how strongly they identify with the respective party (Independents are excluded from this part of the analysis). For measuring trust in government we use the question “How much do you trust people from these groups? [...] The government.” The question is asked on a qualitative scale and coded from 0 to 100 points. We did a median split (34 points) to identify people with high (1) and low (0) levels of trust in government in our sample and create the binary variable *Trust_Gov*∈[0, 1]. To measure autonomy, we use the responses to a battery of nine questions answered on a 5-point Likert scale adapted from [51]. We code people with scores above the median (3.89 points) as having high autonomy and those with values less or equal as having low autonomy, *High_Aut*∈[0, 1]. To measure whether people believe others to have autonomy, we use the question “I think that others feel free to live their life without external interferences”. The question is again coded on a 100-point scale and we do a median split (62 points) to determine low (0) and high (1) values of this belief (*Others_Aut*∈[0, 1]). Finally, to measure the belief in equality of opportunities, we ask participants to rate their agreement with the statement “In the US, everyone has a chance to make it and be successful” on a qualitative scale that is coded from 0 to 100 points. We define those below the median (58 points) as having a low belief and those above as having a high belief in equality of opportunities, *Equal_Opp*∈[0, 1].

S1 to S6 Fig (and the accompanying S11 to S17 Tables) show the marginal effects of each treatment on our dependent variables (as in Tables 3 and 4) for each of the above subgroups. We observe that across all covariates, the effects of the treatments on policy preferences are generally not statistically significant. All of the (unsystematic but) statistically significant effects that we observe for a few variables disappear when correcting for multiple hypothesis testing. Note that the only statistically significant effects (*p* < .05) are: (1) people with low levels of perceived autonomy are more likely to believe there should be increases in private aid when they are incentivized to think more about the true levels of inequality; (2) people with low levels of trust in government are more likely to donate money to a charity (though not more money on average); (3) people in the highest income quintile donate more money on average if they are in *Info_Highest_1%* and people in the two highest income quintiles donate more money in *Info_Lowest_50%* and are more supportive of private redistribution in *Info_Highest_1%*; (4) people with high beliefs in equality of opportunities reduce their perception of fairness in all treatments and increase support for government aid to the poor in *Info_Highest_1%*; (5) Independents and Democrats are more likely to support more government aid to the poor in *Info_Highest_1%* than Republicans; (6) people in mid income ranges are more likely to support government aid to the poor in *Info_Highest_1%*.

A look at the correlations between the moderator variables and inequality beliefs and policy preferences, respectively, shows that all moderators except for *Trust_Gov* and *Own_Aut* correlate significantly with at least one of the inequality beliefs and all of them show significant correlations with at least some of the outcome variables, see S3 Table. This indicates that income, partisanship, trust in government and autonomy beliefs—as discussed in previous work—clearly matter for either perceptions of inequality, fairness preferences or attitudes towards redistribution. However, we do not find a stronger or weaker causal relationship between inequality beliefs and policy views among people who differ with respect to these characteristics.

**Result 3.** *The result that perceived inequality does not have a statistically significant causal effect on fairness views, preferences for redistribution and the willingness to donate to charity holds for all subgroups. In particular, own household income, partisanship, perceived autonomy, perceived differences in autonomy, perceived equality of opportunity and trust in government do not predict differences in this relationship.*

## 4 Discussion

Using a survey experiment, we study how perceived income inequality affects US American’s views about the fairness of inequality and their support for public and private redistribution. We find no statistically significant effect of perceived inequality on policy views or behavior: There is no evidence for fairness views, stated support for public and private redistribution, and the willingness to donate to a charity being causally influenced by beliefs about the income shares received by income earners at the top nor by those at the bottom of the US income distribution. Furthermore, own income, partisanship, trust in government, and perceived personal autonomy and equality of opportunity do not predict how strongly a person’s perception of inequality impacts her policy views in our study. We find precisely estimated null effects over a wide range of specifications and show that isolated significant results disappear when controlling for multiple hypothesis testing.

Not in line with the previous literature on misperceptions of inequality in the US [16, e.g.,] is the observation that US Americans have highly inflated beliefs about the income share earned by the top 1% (however, note that this result is consistent with the findings in [25]). While we can only speculate about the reasons, we believe it is likely that an emphasis on this group in discussions around inequality in political discourse and in the media may have led to a growing awareness about differences in income, especially of those at the very top, in recent years. Note, however, that most previous surveys eliciting beliefs about inequality did not specifically ask about the top income earners and beliefs about this part of the distribution have thus not been as visible. Our elicitation method allows to observe such beliefs and indicate that general conclusions such as “people generally underestimate inequality” may need to be refined and contextualized.

Future studies should complement and expand the way we experimentally manipulate and assess perceived inequality. By asking about the share of total income received by each group, we ask people how income is distributed in the population. This way of assessing the income distribution considers the magnitudes in income differences between groups and assesses income inequality—providing an advantage over related methods such as asking about the share of people in different income groups. In addition, we manipulate information regarding the share of income obtained by groups at the bottom as well as at the top of the distribution, thereby investigating how beliefs about poverty versus beliefs about the affluence of top income earners might affect policy views. Still, by measuring perceived income inequality as the perceived income distribution at the national level, we might not fully capture an average citizen’s complete perception of inequality in their society. In future studies, it might be interesting to assess people’s perception of the incomes of more proximate social groups such as people with the same ethnicity, education level, or gender, or of people in their neighborhood (see also [42,62]). Furthermore, differences in wealth between social groups are much larger than differences in income, i.e., wealth is much less equally distributed in the US [63, e.g.,]. Respondents might have thought about wealth and income together while answering our questions about income shares. Thus, we consider it important that future studies on perceptions of inequality ask jointly about beliefs about how income and wealth are distributed in order to fully understand the relationship between these two perceptions.

Our results add to a growing body of literature on the effect of providing information not only on preferences for redistribution [e.g., 32], but on policy views and political behavior in general (for a review see [64]). They are in line with a few studies that point out that correcting misinformation may indeed affect people’s factual beliefs but preferences may not be conditional on factual beliefs. If it indeed holds true that policy preferences are not conditional on beliefs, we need different types of explanations about which we can only speculate. Fundamental characteristics that more directly influence policy preferences may be party ideology or values such as personal responsibility and meritocratic preferences, which are rather independent of the current economic and social environment (at least in the short term). In addition, we suspect that personal life experience and experiences of people in one’s surroundings may play a role. In order to affect not only people’s perception of the extend of inequality but their redistributive preferences as well, interventions relying upon narratives may be worth exploring in the future.

Our study sheds light on the question how people’s (mis-)perceptions of inequality may translate into a demand for more progressive taxation and redistribution. The results show no evidence that informing people about the extent of inequality in society can effectively alter their support for redistributive policies. Political campaigns aiming at increasing awareness for the need for welfare interventions and more progressive taxation might want to refrain from focusing too much on information about the high incomes of top income earners since, while it is important that citizens are aware of such facts, it may have limited effects on their policy support. Future studies should take on the task of exploring the fundamentals underlying policy preferences in greater detail since beliefs about the social and economic environment seem to have a rather limited explanatory power.

## Supporting information

Data file and code as well as the complete survey instrument: https://doi.org/10.17605/OSF.IO/YKXDR.

S1 Table The surveyed sample compared to the US population. (PDF)

S2 Table Correlation coefficients: inequality beliefs and policy views, donations. (PDF)

S3 Table Correlation coefficients: moderators and policy views, donations, inequality beliefs. (PDF)

S4 Table Estimation as in Table 3: treatments on changes in Fairness, Taxes_Rich, Government_Welfare, Donations_Rich and Private_Welfare with p-values and [60] sharpened q-values. (PDF)

S5 Table Treatments on changes in Fairness, Taxes_Rich, Government_Welfare, Donations_Rich and Private_Welfare for participants who are asked the relevant question immediately after treatment (as first question in ‘Policy Views 2’). (PDF)

S6 Table Treatments on changes in Fairness, Taxes_Rich, Government_Welfare, Donations_Rich and Private_Welfare for participants that never failed an attention check. (PDF)

S7 Table IV results on changes in Fairness, Taxes_Rich, Government_Welfare, Donations_Rich and Private_Welfare, Info_Highest_1% treatment. (PDF)

S8 Table IV results on changes in Fairness, Taxes_Rich, Government_Welfare, Donations_Rich and Private_Welfare, Info_Lowest_50% treatment. (PDF)

S9 Table IV results on changes in Fairness, Taxes_Rich, Government_Welfare, Donations_Rich and Private_Welfare, Monetary_Incentive treatment. (PDF)

S10 Table IV results on changes in Fairness, Taxes_Rich, Government_Welfare, Donations_Rich and Private_Welfare, Monetary_Incentive treatment. (PDF)

S11 Table Marginal effect of treatments by income quintile on changes in Fairness, Taxes_Rich, Government_Welfare, Donations_Rich and Private_Welfare between part 1 and 2, estimated via OLS with p-values and [60] sharpened q-values. (PDF)

S12 Table Marginal effect of treatments by partisanship on changes in Fairness, Taxes_Rich, Government_Welfare, Donations_Rich and Private_Welfare between part 1 and 2, estimated via OLS with p-values and [60] sharpened q-values. (PDF)

S13 Table Marginal effect of treatments by partisanship on changes in support for redistribution measured by Fairness, Taxes_Rich, Government_Welfare, Donations_Rich and Private_Welfare estimated via OLS with p-values and [60] sharpened q-values. Continued.
(PDF)

S14 Table Marginal effect of treatments for high vs low trust in government on changes in support for redistribution measured by Fairness, Taxes_Rich, Government_Welfare, Donations_Rich and Private_Welfare estimated via OLS with p-values and [60] sharpened q-values. (PDF)

S15 Table Marginal effect of treatments for high vs low perceived own autonomy on changes in support for redistribution measured by Fairness, Taxes_Rich, Government_Welfare, Donations_Rich and Private_Welfare estimated via OLS with p-values and [60] sharpened q-values. (PDF)

S16 Table Marginal effect of treatments for high vs low perceived autonomy of others on changes in support for redistribution measured by Fairness, Taxes_Rich, Government_Welfare, Donations_Rich and Private_Welfare estimated via OLS with p-values and [60] sharpened q-values. (PDF)

S17 Table Marginal effect of treatments for high vs low belief in equal opportunity on changes in support for redistribution measured by Fairness, Taxes_Rich, Government_Welfare, Donations_Rich and Private_Welfare estimated via OLS with p-values and [60] sharpened q-values. (PDF)

S18 Table Treatments on changes in Fairness, Taxes_Rich, Government_Welfare, Donations_Rich and Private_Welfare in October and November. (PDF)

S1 Fig Marginal Effects of Treatment Info_Lowest_50%, Info_Highest_1% and Monetary_Incentive by Income Quintiles. OLS with robust standard errors. Panels show changes in Fairness, Taxes_Rich, Government_Welfare, Donations_Rich, and Private_Welfare, as well as Donation_Decision and Amount_Donated.
(PDF)

S2 Fig Marginal Effects of treatment Info_Lowest_50%, Info_Highest_1% and Monetary_Incentive by Partisanship. OLS with robust standard errors. Panels show changes in Fairness, Taxes_Rich, Government_Welfare, Donations_Rich, and Private_Welfare, as well as Donation_Decision and Amount_Donated.
(PDF)

S3 Fig Marginal Effects of treatment Info_Lowest_50%, Info_Highest_1% and Monetary_Incentive by Trust in Government. OLS with robust standard errors. Panels show changes in Fairness, Taxes_Rich, Government_Welfare, Donations_Rich, and Private_Welfare, as well as Donation_Decision and Amount_Donated.
(PDF)

S4 Fig Marginal Effects of treatment Info_Lowest_50%, Info_Highest_1% and Monetary_Incentive by Autonomy. OLS with robust standard errors. Panels show changes in Fairness, Taxes_Rich, Government_Welfare, Donations_Rich, and Private_Welfare, as well as Donation_Decision and Amount_Donated.
(PDF)

S5 Fig Marginal Effects of treatment Info_Lowest_50%, Info_Highest_1% and Monetary_Incentive by Autonomy of Others. OLS with robust standard errors. Panels show changes in Fairness, Taxes_Rich, Government_Welfare, Donations_Rich, and Private_Welfare, as well as Donation_Decision and Amount_Donated.
(PDF)

S6 Fig Marginal Effects of treatment Info_Lowest_50%, Info_Highest_1% and Monetary_Incentive by Belief in Equal Opportunity. OLS with robust standard errors. Panels show changes in Fairness, Taxes_Rich, Government_Welfare, Donations_Rich, and Private_Welfare, as well as Donation_Decision and Amount_Donated.
(PDF)
