# Supplementary material for: On the relationship of perceived inequality and policy preferences
Source: PLoS One. 2026 Aug 19;21(8):e0341298. doi: 10.1371/journal.pone.0341298 (PMC13489463; doi:10.1371/journal.pone.0341298)
Supplement: Supplementary file 1 [file pone.0341298.s001.pdf]

S1 Table. The surveyed sample compared to the US population.

		Sample	US population
Age	18-29	21.89%	20.9%
	30-50	33.17%	33.25%
	50 and older	44.93%	45.86%
Gender	Female	54.37%	51.27%
Race	Non-hispanic White	77.06%	63.14%
Income	<50.000 USD	41.67% (33.27%)	29%
	50,000-130,000 USD	45.80% (52.19%)	52%
	>130.000 USD	12.53% (14.54%)	19%

Population data (age, gender, race) is taken from the 2019 US census for the adult population (18 years and older, which is also the restriction to enter our survey). Data on annual household income from the Pew research center (2018). The income categories are defined as follows: A middle class income is defined as earning more than 2/3 of the median income (i.e. 45.200USD) and less than twice the median income (i.e. 135.600USD) in 2018. These are the thresholds for a three-person household; the average householdsize in our sample is 2.67. At the expense of accuracy, we rounded the thresholds to 50,000 and 130,000 when asking about income in the survey. The shares of three-person households in each category is reported in brackets (571 respondents). Link: https://www.pewresearch.org/fact-tank/2018/09/06/the-american-middle-class-is-stable-in-size-but-losing-ground-financially-to-upper-income-families/.
