# Supplementary material for: On the relationship of perceived inequality and policy preferences
Source: PLoS One. 2026 Aug 19;21(8):e0341298. doi: 10.1371/journal.pone.0341298 (PMC13489463; doi:10.1371/journal.pone.0341298)
Supplement: Supplementary file 2 [file pone.0341298.s002.pdf]

S2 Table. Correlation coefficients: inequality beliefs and policy views, donations.

	Fairness	Taxes_Rich	Gov_Welfare	Donations_Rich	Private_Welfare	Donation
Belief_Low_Inc	.229*<br>(.0000)	-.135*<br>(.0003)	-.056<br>(.1356)	-.033<br>(.3744)	-.087*<br>(.0202)	-.019<br>(.6190)
Belief_High_Inc	-.189*<br>(.0000)	.135*<br>(.0003)	.075*<br>(.0454)	.050<br>(.1793)	.078*<br>(.0369)	.074*<br>(.0464)

Fairness and political views are part 1 variables. Pairwise correlation coefficients with significance levels. Data from treatment Control (717 observations).
