# Supplementary material for: On the relationship of perceived inequality and policy preferences
Source: PLoS One. 2026 Aug 19;21(8):e0341298. doi: 10.1371/journal.pone.0341298 (PMC13489463; doi:10.1371/journal.pone.0341298)
Supplement: Supplementary file 3 [file pone.0341298.s003.pdf]

S3 Table. Correlation coefficients: moderators and policy views, donations, inequality beliefs.

	Fairness	Taxes_Rich	Gov_Welfare	Don_Rich	Priv_Welfare	Donation	Belief_Low_Inc	Belief_High_Inc
Income	.182*<br>(.0000)	-.049<br>(.1870)	-.145*<br>(.0001)	-.033<br>(.3767)	-.002<br>(.9628)	.054<br>(.1478)	-.077*<br>(.0390)	.068<br>(.0686)
Partisan	.571*<br>(.0000)	-.499*<br>(.0000)	-.489*<br>(.0000)	-.339*<br>(.0000)	-.182*<br>(.0000)	-.092*<br>(.0136)	.185*<br>(.0000)	-.155*<br>(.0000)
Trust_Gov	.245*<br>(.0000)	-.032<br>(.3933)	.021<br>(.5830)	-.085*<br>(.0224)	-.02<br>(.5994)	.599<br>(.1143)	.034<br>(.3652)	-.032<br>(.3864)
Own_Aut	-.012<br>(.7540)	-.013<br>(.7214)	-.020<br>(.5940)	-.025<br>(.5103)	-.019<br>(.6163)	.091*<br>(.0159)	-.056<br>(.1381)	.042<br>(.2659)
Others_Aut	.117*<br>(.0017)	.036<br>(.3409)	.039<br>(.2996)	.018<br>(.6273)	.051<br>(.1689)	.005<br>(.8845)	.201*<br>(.0000)	-.088*<br>(.0190)
Equal_Opp	.594*<br>(.0000)	-.425*<br>(.0000)	-.427*<br>(.0000)	-.276*<br>(.0000)	-.175*<br>(.0000)	-.017<br>(.6460)	.211*<br>(.0000)	-.141*<br>(.0001)

Fairness and political views are part 1 variables. Pairwise correlation coefficients with significance levels. Data from treatment Control (717 observations).
