# Supplementary material for: On the relationship of perceived inequality and policy preferences
Source: PLoS One. 2026 Aug 19;21(8):e0341298. doi: 10.1371/journal.pone.0341298 (PMC13489463; doi:10.1371/journal.pone.0341298)
Supplement: Supplementary file 4 [file pone.0341298.s004.pdf]

S4 Table. Estimation as in Table 3: treatments on changes in Fairness, Taxes_Rich, Government_Welfare, Donations_Rich and Private_Welfare with p-values and Anderson (2008)sh arpened q-values.

	Fairness	Taxes Rich	Government Welfare	Donations Rich	Private Welfare
Info_Lowest_50% × Post-Treatment	-1.227	-0.222	0.126	0.519	0.612
p-value	0.1	0.699	0.816	0.444	0.306
sharpened q-value	1.000	1.000	1.000	1.000	1.000
Info_Highest_1% × Post-Treatment	-0.824	0.109	1.207	-0.496	0.934
p-value	0.24	0.856	0.025	0.453	0.141
sharpened q-value	0.471	0.667	0.143	0.545	0.393
Monetary_Incentive × Post-Treatment	-1.029	-0.307	0.294	-0.032	1.058
p-value	0.149	0.627	0.624	0.962	0.116
sharpened q-value	0.594	0.888	0.888	1.000	0.594
N	5764	5764	5764	5764	5764
