# Supplementary material for: On the relationship of perceived inequality and policy preferences
Source: PLoS One. 2026 Aug 19;21(8):e0341298. doi: 10.1371/journal.pone.0341298 (PMC13489463; doi:10.1371/journal.pone.0341298)
Supplement: Supplementary file 5 [file pone.0341298.s005.pdf]

S5 Table. Treatments on changes in Fairness, Taxes_Rich, Government_Welfare, Donations_Rich and Private_Welfare for participants who are asked the relevant question immediately after treatment (as first question in 'Policy Views 2').

	Fairness	Taxes Rich	Government Welfare	Donations Rich	Private Welfare
Info_Lowest_50%	-5.842**	3.540*	3.129	-2.021	-2.397
	(2.663)	(2.043)	(2.231)	(1.760)	(1.693)
Info_Highest_1%	-5.628**	2.884	2.163	.284	1.153
	(2.734)	(2.074)	(2.245)	(1.650)	(1.785)
Monetary_Incentive	-2.413	3.200	2.995	-2.053	-1.150
	(2.834)	(1.962)	(2.212)	(1.704)	(1.813)
Post-Treatment	-1.278	.707	1.522***	.208	1.239*
	(1.041)	(.784)	(.585)	(.716)	(.670)
Info_Lowest_50% × Post-Treatment	-1.750	.118	-.150	1.413	-.790
	(1.382)	(.941)	(.839)	(1.333)	(1.016)
Info_Highest_1% × Post-Treatment	-.623	.907	1.145	-.486	-1.199
	(1.291)	(1.074)	(.925)	(1.043)	(.972)
Monetary_Incentive × Post-Treatment	-.038	.779	.114	.565	-.305
	(1.358)	(1.027)	(.916)	(1.225)	(1.108)
Constant	34.387***	78.901***	69.211***	81.482***	72.063***
	(2.062)	(1.473)	(1.622)	(1.121)	(1.186)
N	1926	1908	1908	1930	1930

Robust standard errors clustered at the individual level in parentheses.

* $p < 0.1$, ** $p < 0.05$, *** $p < 0.01$
