# Supplementary material for: On the relationship of perceived inequality and policy preferences
Source: PLoS One. 2026 Aug 19;21(8):e0341298. doi: 10.1371/journal.pone.0341298 (PMC13489463; doi:10.1371/journal.pone.0341298)
Supplement: Supplementary file 6 [file pone.0341298.s006.pdf]

S6 Table. Treatments on changes in Fairness, Taxes_Rich, Government_Welfare, Donations_Rich and Private_Welfare for participants that never failed an attention check.

	Fairness	Taxes Rich	Government Welfare	Donations Rich	Private Welfare
Info_Lowest_50%	-2.129	2.347*	2.091	.796	1.203
	(1.688)	(1.207)	(1.349)	(1.103)	(1.091)
Info_Highest_1%	-2.564	1.630	1.135	1.807*	2.174**
	(1.675)	(1.241)	(1.368)	(1.076)	(1.100)
Monetary_Incentive	-.197	.838	.336	.158	.791
	(1.693)	(1.231)	(1.397)	(1.098)	(1.137)
Post-Treatment	-2.176***	.671	1.016***	.436	.407
	(.512)	(.488)	(.384)	(.512)	(.464)
Info_Lowest_50% × Post-Treatment	-.797	-.120	.304	.265	.136
	(.790)	(.614)	(.569)	(.732)	(.633)
Info_Highest_1% × Post-Treatment	-.264	-.317	1.235**	-.444	.613
	(.722)	(.616)	(.569)	(.681)	(.668)
Monetary_Incentive × Post-Treatment	-.689	-.131	.349	-.318	.569
	(.740)	(.679)	(.624)	(.718)	(.714)
Constant	31.942***	80.052***	70.409***	78.971***	69.994***
	(1.219)	(.885)	(.990)	(.770)	(.796)
N	5022	5022	5022	5022	5022

Robust standard errors clustered at the individual level in parentheses.

* $p < 0.1$, ** $p < 0.05$, *** $p < 0.01$
