# Supplementary material for: On the relationship of perceived inequality and policy preferences
Source: PLoS One. 2026 Aug 19;21(8):e0341298. doi: 10.1371/journal.pone.0341298 (PMC13489463; doi:10.1371/journal.pone.0341298)
Supplement: Supplementary file 7 [file pone.0341298.s007.pdf]

S7 Table. IV results on changes in Fairness, Taxes_Rich, Government_Welfare, Donations_Rich and Private_Welfare, Info_Highest_1% treatment.

	Fairness	Taxes Rich	Government Welfare	Donations Rich	Private Welfare
Within 5 percentage points of true income for top 1 percent	-1.964	.260	2.877**	-1.181	2.226
	(1.668)	(1.429)	(1.291)	(1.572)	(1.515)
N	1439	1439	1439	1439	1439

Robust standard errors in parentheses.

* $p < 0.1$, ** $p < 0.05$, *** $p < 0.01$
