# Supplementary material for: On the relationship of perceived inequality and policy preferences
Source: PLoS One. 2026 Aug 19;21(8):e0341298. doi: 10.1371/journal.pone.0341298 (PMC13489463; doi:10.1371/journal.pone.0341298)
Supplement: Supplementary file 8 [file pone.0341298.s008.pdf]

S8 Table. IV results on changes in Fairness, Taxes_Rich, Government_Welfare, Donations_Rich and Private_Welfare, Info_Lowest_50% treatment.

	Fairness	Taxes Rich	Government Welfare	Donations Rich	Private Welfare
Within 5 percentage points of true income for lowest 50 percent	-3.957	-.717	.406	1.675	1.973
	(2.422)	(1.851)	(1.745)	(2.186)	(1.933)
N	1437	1437	1437	1437	1437

Robust standard errors in parentheses.

* $p < 0.1$, ** $p < 0.05$, *** $p < 0.01$
