# Supplementary material for: On the relationship of perceived inequality and policy preferences
Source: PLoS One. 2026 Aug 19;21(8):e0341298. doi: 10.1371/journal.pone.0341298 (PMC13489463; doi:10.1371/journal.pone.0341298)
Supplement: Supplementary file 9 [file pone.0341298.s009.pdf]

**S9 Table. IV results on changes in Fairness, Taxes_Rich, Government_Welfare, Donations_Rich and Private_Welfare, Monetary_Incentive treatment.**

| | Fairness | Taxes Rich | Government Welfare | Donations Rich | Private Welfare |
|---|---|---|---|---|---|
| Within 5 percentage points of true income for top 1 percent | -24.921 | -7.437 | 7.114 | -.774 | 25.634 |
| | (18.453) | (15.557) | (14.703) | (16.299) | (17.595) |
| N | 1440 | 1440 | 1440 | 1440 | 1440 |

Robust standard errors in parentheses.

* $p < 0.1$, ** $p < 0.05$, *** $p < 0.01$
