# Supplementary material for: On the relationship of perceived inequality and policy preferences
Source: PLoS One. 2026 Aug 19;21(8):e0341298. doi: 10.1371/journal.pone.0341298 (PMC13489463; doi:10.1371/journal.pone.0341298)
Supplement: Supplementary file 10 [file pone.0341298.s010.pdf]

S10 Table. IV results on changes in Fairness, Taxes_Rich, Government_Welfare, Donations_Rich and Private_Welfare, Monetary_Incentive treatment.

	Fairness	Taxes Rich	Government Welfare	Donations Rich	Private Welfare
Within 5 percentage points of true income for lowest 50 percent	-26.091	-7.786	7.448	-.811	26.838
	(23.486)	(16.654)	(15.779)	(17.097)	(23.310)
N	1440	1440	1440	1440	1440

Robust standard errors in parentheses.

* $p < 0.1$, ** $p < 0.05$, *** $p < 0.01$
