# Supplementary material for: On the relationship of perceived inequality and policy preferences
Source: PLoS One. 2026 Aug 19;21(8):e0341298. doi: 10.1371/journal.pone.0341298 (PMC13489463; doi:10.1371/journal.pone.0341298)
Supplement: Supplementary file 11 [file pone.0341298.s011.pdf]

S11 Table. Marginal effect of treatments by income quintile on changes in Fairness, Taxes_Rich, Government_Welfare, Donations_Rich and Private_Welfare between part 1 and 2, estimated via OLS with p-values and Anderson (2008) sharpened q-values.

	Fairness	Taxes Rich	Government Welfare	Donations Rich	Private Welfare
**Info_Lowest_50%**					
Marginal Effect for Income $< \$25,000$	-2.77	-0.18	0.08	0.33	0.45
p-value	0.06	0.87	0.94	0.80	0.66
sharpened q-value	1.00	1.00	1.00	1.00	1.00
Marginal Effect for Income $25,000-$50,000	-1.95	-0.21	0.10	0.42	0.52
p-value	0.05	0.78	0.88	0.64	0.46
sharpened q-value	1.00	1.00	1.00	1.00	1.00
Marginal Effect for Income $50,000-$80,000	-1.14	-0.23	0.13	0.51	0.60
p-value	0.13	0.69	0.81	0.45	0.32
sharpened q-value	1.00	1.00	1.00	1.00	1.00
Marginal Effect for Income $80,000-$130,000	-0.32	-0.26	0.15	0.59	0.67
p-value	0.73	0.72	0.81	0.47	0.42
sharpened q-value	1.00	1.00	1.00	1.00	1.00
Marginal Effect for Income $> \$130,000$	0.50	-0.28	0.17	0.68	0.74
p-value	0.72	0.79	0.85	0.57	0.54
sharpened q-value	1.00	1.00	1.00	1.00	1.00
**T_Info_Highest_1%**					
Marginal Effect for Income $< \$25,000$	-0.89	-0.67	1.23	-1.68	-1.21
p-value	0.50	0.55	0.21	0.16	0.27
sharpened q-value	1.00	1.00	1.00	1.00	1.00
Marginal Effect for Income $25,000-$50,000	-0.85	-0.26	1.22	-1.07	-0.08
p-value	0.34	0.73	0.07	0.20	0.91
sharpened q-value	1.00	1.00	1.00	1.00	1.00
Marginal Effect for Income $50,000-$80,000	-0.82	0.15	1.20	-0.45	1.04
p-value	0.25	0.80	0.02	0.49	0.10
sharpened q-value	1.00	1.00	1.00	1.00	1.00
Marginal Effect for Income $80,000-$130,000	-0.78	0.56	1.19	0.16	2.17
p-value	0.40	0.47	0.06	0.85	0.01
sharpened q-value	1.00	1.00	1.00	1.00	0.70
Marginal Effect for Income $> \$130,000$	-0.75	0.97	1.18	0.78	3.29
p-value	0.59	0.40	0.19	0.52	0.01
sharpened q-value	1.00	1.00	1.00	1.00	0.70
**T_Monetary_Incentive**					
Marginal Effect for Income $< \$25,000$	0.00	-0.47	-0.30	-0.73	0.47
p-value	1.00	0.69	0.78	0.55	0.69
sharpened q-value	1.00	1.00	1.00	1.00	1.00
Marginal Effect for Income $25,000-$50,000	-0.55	-0.39	0.01	-0.38	0.77
p-value	0.55	0.62	0.98	0.65	0.33
sharpened q-value	1.00	1.00	1.00	1.00	1.00
Marginal Effect for Income $50,000-$80,000	-1.10	-0.31	0.33	-0.02	1.06
p-value	0.12	0.63	0.59	0.98	0.12
sharpened q-value	1.00	1.00	1.00	1.00	1.00
Marginal Effect for Income $80,000-$130,000	-1.64	-0.22	0.65	0.34	1.35
p-value	0.07	0.80	0.49	0.71	0.16
sharpened q-value	1.00	1.00	1.00	1.00	1.00
Marginal Effect for Income $> \$130,000$	-2.19	-0.14	0.97	0.69	1.65
p-value	0.10	0.91	0.49	0.60	0.24
sharpened q-value	1.00	1.00	1.00	1.00	1.00
