# Supplementary material for: On the relationship of perceived inequality and policy preferences
Source: PLoS One. 2026 Aug 19;21(8):e0341298. doi: 10.1371/journal.pone.0341298 (PMC13489463; doi:10.1371/journal.pone.0341298)
Supplement: Supplementary file 12 [file pone.0341298.s012.pdf]

S12 Table. Marginal effect of treatments by partisanship on changes in Fairness, Taxes_Rich, Government_Welfare, Donations_Rich and Private_Welfare between part 1 and 2, estimated via OLS with p-values and Anderson (2008) sharpened q-values.

	Fairness	Taxes Rich	Government Welfare	Donations Rich	Private Welfare
**Info_Lowest_50%**					
Marginal Effect for Strong Republicans	-0.56	-0.23	0.06	0.94	-0.34
p-value	0.58	0.77	0.94	0.37	0.69
sharpened q-value	1.00	1.00	1.00	1.00	1.00
Marginal Effect for Weak Republicans	-0.85	-0.22	0.10	0.71	0.06
p-value	0.28	0.72	0.87	0.40	0.93
sharpened q-value	1.00	1.00	1.00	1.00	1.00
Marginal Effect for Leaning Republicans	-1.15	-0.22	0.13	0.48	0.45
p-value	0.11	0.70	0.81	0.49	0.44
sharpened q-value	1.00	1.00	1.00	1.00	1.00
Marginal Effect for Independents	-1.44	-0.21	0.17	0.25	0.85
p-value	0.10	0.74	0.77	0.70	0.21
sharpened q-value	1.00	1.00	1.00	1.00	1.00
Marginal Effect for Leaning Democrats	-1.74	-0.21	0.20	0.02	1.25
p-value	0.12	0.80	0.78	0.98	0.15
sharpened q-value	1.00	1.00	1.00	1.00	1.00
Marginal Effect for Weak Democrats	-2.03	-0.20	0.24	-0.21	1.64
p-value	0.16	0.85	0.79	0.83	0.14
sharpened q-value	1.00	1.00	1.00	1.00	1.00
Marginal Effect for Strong Democrats	-2.33	-0.19	0.28	-0.44	2.04
p-value	0.20	0.88	0.81	0.71	0.14
sharpened q-value	1.00	1.00	1.00	1.00	1.00
**T_Info_Highest_1%**					
Marginal Effect for Strong Republicans	-0.44	-0.21	0.23	0.02	0.58
p-value	0.62	0.80	0.75	0.99	0.51
sharpened q-value	1.00	1.00	1.00	1.00	1.00
Marginal Effect for Weak Republicans	-0.60	-0.07	0.65	-0.21	0.73
p-value	0.39	0.91	0.25	0.78	0.30
sharpened q-value	1.00	1.00	1.00	1.00	1.00
Marginal Effect for Leaning Republicans	-0.76	0.07	1.08	-0.44	0.87
p-value	0.25	0.91	0.04	0.51	0.16
sharpened q-value	1.00	1.00	1.00	1.00	1.00
Marginal Effect for Independents	-0.93	0.20	1.50	-0.66	1.01
p-value	0.25	0.76	0.01	0.35	0.15
sharpened q-value	1.00	1.00	1.00	1.00	1.00
Marginal Effect for Leaning Democrats	-1.09	0.34	1.92	-0.89	1.15
p-value	0.30	0.68	0.01	0.32	0.20
sharpened q-value	1.00	1.00	1.00	1.00	1.00
Marginal Effect for Weak Democrats	-1.26	0.48	2.35	-1.12	1.29
p-value	0.35	0.65	0.02	0.32	0.26
sharpened q-value	1.00	1.00	1.00	1.00	1.00
Marginal Effect for Strong Democrats	-1.42	0.62	2.77	-1.34	1.44
p-value	0.40	0.64	0.03	0.34	0.32
sharpened q-value	1.00	1.00	1.00	1.00	1.00
