# Supplementary material for: On the relationship of perceived inequality and policy preferences
Source: PLoS One. 2026 Aug 19;21(8):e0341298. doi: 10.1371/journal.pone.0341298 (PMC13489463; doi:10.1371/journal.pone.0341298)
Supplement: Supplementary file 13 [file pone.0341298.s013.pdf]

S13 Table. Marginal effect of treatments by partisanship on changes in support for redistribution measured by Fairness, Taxes_Rich, Government_Welfare, Donations_Rich and Private_Welfare estimated via OLS with p-values and Anderson (2008) sharpened q-values. Continued.

	Fairness	Taxes Rich	Government Welfare	Donations Rich	Private Welfare
**Monetary_Incentive**					
Marginal Effect for Strong Republicans	-1.10	0.70	0.22	-0.65	0.76
p-value	0.26	0.44	0.79	0.52	0.44
sharpened q-value	1.00	1.00	1.00	1.00	1.00
Marginal Effect for Weak Republicans	-1.06	0.26	0.25	-0.39	0.87
p-value	0.17	0.72	0.69	0.63	0.26
sharpened q-value	1.00	1.00	1.00	1.00	1.00
Marginal Effect for Leaning Republicans	-1.02	-0.19	0.29	-0.13	0.98
p-value	0.14	0.77	0.62	0.85	0.14
sharpened q-value	1.00	1.00	1.00	1.00	1.00
Marginal Effect for Independents	-0.98	-0.63	0.32	0.13	1.10
p-value	0.22	0.37	0.63	0.85	0.12
sharpened q-value	1.00	1.00	1.00	1.00	1.00
Marginal Effect for Leaning Democrats	-0.95	-1.07	0.36	0.39	1.21
p-value	0.36	0.22	0.68	0.64	0.17
sharpened q-value	1.00	1.00	1.00	1.00	1.00
Marginal Effect for Weak Democrats	-0.91	-1.52	0.39	0.65	1.33
p-value	0.49	0.18	0.72	0.53	0.23
sharpened q-value	1.00	1.00	1.00	1.00	1.00
Marginal Effect for Strong Democrats	-0.87	-1.96	0.43	0.91	1.44
p-value	0.60	0.16	0.75	0.48	0.30
sharpened q-value	1.00	1.00	1.00	1.00	1.00
