# Supplementary material for: On the relationship of perceived inequality and policy preferences
Source: PLoS One. 2026 Aug 19;21(8):e0341298. doi: 10.1371/journal.pone.0341298 (PMC13489463; doi:10.1371/journal.pone.0341298)
Supplement: Supplementary file 14 [file pone.0341298.s014.pdf]

S14 Table. Marginal effect of treatments for high vs low trust in government on changes in support for redistribution measured by Fairness, Taxes_Rich, Government_Welfare, Donations_Rich and Private_Welfare estimated via OLS with p-values and Anderson (2008) sharpened q-values.

	Fairness	Taxes Rich	Government Welfare	Donations Rich	Private Welfare
**Info_Lowest_50%**					
Marginal Effect for Low Trust in Government	-1.17	0.33	-0.14	0.52	1.13
p-value	0.24	0.67	0.85	0.58	0.20
sharpened q-value	1.00	1.00	1.00	1.00	1.00
Marginal Effect for High Trust in Government	-1.28	-0.79	0.45	0.54	0.14
p-value	0.25	0.36	0.56	0.58	0.86
sharpened q-value	1.00	1.00	1.00	1.00	1.00
**T_Info_Highest_1%**					
Marginal Effect for Low Trust in Government	0.26	0.18	1.01	-0.21	1.55
p-value	0.78	0.83	0.15	0.81	0.10
sharpened q-value	1.00	1.00	1.00	1.00	1.00
Marginal Effect for High Trust in Government	-1.94	0.06	1.42	-0.78	0.34
p-value	0.06	0.95	0.08	0.44	0.70
sharpened q-value	1.00	1.00	1.00	1.00	1.00
**T_Monetary_Incentive**					
Marginal Effect for Low Trust in Government	-0.75	0.98	0.42	0.28	1.13
p-value	0.42	0.27	0.58	0.76	0.28
sharpened q-value	1.00	1.00	1.00	1.00	1.00
Marginal Effect for High Trust in Government	-1.30	-1.52	0.17	-0.34	0.94
p-value	0.23	0.09	0.86	0.73	0.28
sharpened q-value	1.00	1.00	1.00	1.00	1.00
