# Supplementary material for: On the relationship of perceived inequality and policy preferences
Source: PLoS One. 2026 Aug 19;21(8):e0341298. doi: 10.1371/journal.pone.0341298 (PMC13489463; doi:10.1371/journal.pone.0341298)
Supplement: Supplementary file 15 [file pone.0341298.s015.pdf]

S15 Table. Marginal effect of treatments for high vs low perceived own autonomy on changes in support for redistribution measured by Fairness, Taxes_Rich, Government_Welfare, Donations_Rich and Private_Welfare estimated via OLS with p-values and Anderson (2008) sharpened q-values.

	Fairness	Taxes Rich	Government Welfare	Donations Rich	Private Welfare
**Info_Lowest_50%**					
Marginal Effect for Low Autonomy	-1.09	0.57	0.09	1.01	0.47
p-value	0.25	0.41	0.91	0.28	0.54
sharpened q-value	1.00	1.00	1.00	1.00	1.00
Marginal Effect for High Autonomy	-1.88	-1.48	0.06	-0.08	1.13
p-value	0.11	0.14	0.94	0.94	0.22
sharpened q-value	1.00	1.00	1.00	1.00	1.00
**T_Info_Highest_1%**					
Marginal Effect for Low Autonomy	-0.62	0.82	1.37	0.13	1.29
p-value	0.48	0.29	0.07	0.89	0.14
sharpened q-value	1.00	1.00	1.00	1.00	1.00
Marginal Effect for High Autonomy	-1.16	-0.99	0.89	-1.27	0.44
p-value	0.30	0.32	0.22	0.18	0.63
sharpened q-value	1.00	1.00	1.00	1.00	1.00
**T_Monetary_Incentive**					
Marginal Effect for Low Autonomy	-1.61	-0.01	0.33	0.47	1.85
p-value	0.09	0.99	0.72	0.63	0.04
sharpened q-value	1.00	1.00	1.00	1.00	1.00
Marginal Effect for High Autonomy	-0.51	-0.96	0.19	-0.60	0.31
p-value	0.64	0.36	0.77	0.51	0.76
sharpened q-value	1.00	1.00	1.00	1.00	1.00
