# Supplementary material for: On the relationship of perceived inequality and policy preferences
Source: PLoS One. 2026 Aug 19;21(8):e0341298. doi: 10.1371/journal.pone.0341298 (PMC13489463; doi:10.1371/journal.pone.0341298)
Supplement: Supplementary file 16 [file pone.0341298.s016.pdf]

**S16 Table. Marginal effect of treatments for high vs low perceived autonomy of others on changes in support for redistribution measured by Fairness, Taxes_Rich, Government_Welfare, Donations_Rich and Private_Welfare estimated via OLS with p-values and Anderson (2008) sharpened q-values.**

| | Fairness | Taxes Rich | Government Welfare | Donations Rich | Private Welfare |
|---|---|---|---|---|---|
| **Info_Lowest_50%** | | | | | |
| Marginal Effect for Low Belief in Autonomy of Others | -0.44 | -0.48 | 0.22 | 0.06 | 0.55 |
| p-value | 0.64 | 0.49 | 0.74 | 0.95 | 0.48 |
| sharpened q-value | 1.00 | 1.00 | 1.00 | 1.00 | 1.00 |
| Marginal Effect for High Belief in Autonomy of Others | -2.07 | 0.06 | 0.01 | 0.98 | 0.65 |
| p-value | 0.08 | 0.95 | 0.99 | 0.32 | 0.48 |
| sharpened q-value | 1.00 | 1.00 | 1.00 | 1.00 | 1.00 |
| **T_Info_Highest_1%** | | | | | |
| Marginal Effect for Low Belief in Autonomy of Others | -0.55 | 0.51 | 1.42 | -1.06 | -0.06 |
| p-value | 0.55 | 0.45 | 0.06 | 0.23 | 0.94 |
| sharpened q-value | 1.00 | 1.00 | 1.00 | 1.00 | 1.00 |
| Marginal Effect for High Belief in Autonomy of Others | -1.10 | -0.30 | 0.99 | 0.07 | 1.94 |
| p-value | 0.30 | 0.76 | 0.19 | 0.94 | 0.05 |
| sharpened q-value | 1.00 | 1.00 | 1.00 | 1.00 | 1.00 |
| **T_Monetary_Incentive** | | | | | |
| Marginal Effect for Low Belief in Autonomy of Others | -0.76 | -0.74 | 0.38 | -0.15 | 0.61 |
| p-value | 0.44 | 0.30 | 0.63 | 0.87 | 0.53 |
| sharpened q-value | 1.00 | 1.00 | 1.00 | 1.00 | 1.00 |
| Marginal Effect for High Belief in Autonomy of Others | -1.32 | 0.16 | 0.18 | 0.06 | 1.51 |
| p-value | 0.20 | 0.88 | 0.84 | 0.95 | 0.11 |
| sharpened q-value | 1.00 | 1.00 | 1.00 | 1.00 | 1.00 |
