# Supplementary material for: On the relationship of perceived inequality and policy preferences
Source: PLoS One. 2026 Aug 19;21(8):e0341298. doi: 10.1371/journal.pone.0341298 (PMC13489463; doi:10.1371/journal.pone.0341298)
Supplement: Supplementary file 17 [file pone.0341298.s017.pdf]

S17 Table. Marginal effect of treatments for high vs low belief in equal opportunity on changes in support for redistribution measured by Fairness, Taxes_Rich, Government_Welfare, Donations_Rich and Private_Welfare estimated via OLS with p-values and Anderson (2008) sharpened q-values.

	Fairness	Taxes Rich	Government Welfare	Donations Rich	Private Welfare
**Info_Lowest_50%**					
Marginal Effect for Low Belief in Equal Opportunity	0.82	0.32	0.26	0.86	0.69
p-value	0.31	0.67	0.71	0.37	0.36
sharpened q-value	1.00	1.00	1.00	1.00	1.00
Marginal Effect for High Belief in Equal Opportunity	-3.33	-0.78	0.01	0.15	0.52
p-value	0.01	0.37	0.99	0.88	0.58
sharpened q-value	0.32	1.00	1.00	1.00	1.00
**T_Info_Highest_1%**					
Marginal Effect for Low Belief in Equal Opportunity	0.87	-0.26	0.68	-0.64	0.60
p-value	0.26	0.75	0.36	0.47	0.46
sharpened q-value	1.00	1.00	1.00	1.00	1.00
Marginal Effect for High Belief in Equal Opportunity	-2.57	0.52	1.79	-0.36	1.27
p-value	0.03	0.55	0.02	0.71	0.19
sharpened q-value	0.37	1.00	0.37	1.00	1.00
**T_Monetary_Incentive**					
Marginal Effect for Low Belief in Equal Opportunity	0.20	-0.74	0.68	-0.18	1.49
p-value	0.81	0.32	0.39	0.85	0.11
sharpened q-value	1.00	1.00	1.00	1.00	1.00
Marginal Effect for High Belief in Equal Opportunity	-2.22	0.12	-0.08	0.11	0.64
p-value	0.05	0.90	0.93	0.91	0.51
sharpened q-value	0.51	1.00	1.00	1.00	1.00
