# Supplementary material for: On the relationship of perceived inequality and policy preferences
Source: PLoS One. 2026 Aug 19;21(8):e0341298. doi: 10.1371/journal.pone.0341298 (PMC13489463; doi:10.1371/journal.pone.0341298)
Supplement: Supplementary file 18 [file pone.0341298.s018.pdf]

S18 Table. Treatments on changes in Fairness, Taxes_Rich, Government_Welfare, Donations_Rich and Private_Welfare in October and November.

	Fairness	Taxes Rich	Government Welfare	Donations Rich	Private Welfare
Info_Lowest_50%	-.952	-.198	.494	.615	.577
	(.830)	(.655)	(.577)	(.770)	(.643)
Info_Highest_1%	-.906	.421	1.303**	-.289	.645
	(.797)	(.680)	(.592)	(.755)	(.682)
Monetary Incentive	-1.153	-.169	.023	.036	1.050
	(.798)	(.703)	(.678)	(.785)	(.734)
November	-.740	.081	-.056	1.762*	-.656
	(1.159)	(1.152)	(1.015)	(.982)	(1.329)
Info_Lowest_50% × November	-1.371	-.119	-1.842	-.481	.175
	(1.895)	(1.349)	(1.530)	(1.605)	(1.658)
Info_Highest_1% × November	.416	-1.555	-.476	-1.038	1.443
	(1.662)	(1.438)	(1.404)	(1.519)	(1.752)
Monetary Incentive × November	.621	-.689	1.348	-.350	.045
	(1.776)	(1.607)	(1.452)	(1.445)	(1.798)
Constant	-1.707***	.646	1.118***	-.000	.272
	(.581)	(.508)	(.398)	(.564)	(.456)
N	2882	2882	2882	2882	2882

Robust standard errors clustered at the individual level in parentheses.

* $p < 0.1$, ** $p < 0.05$, *** $p < 0.01$
