# Supplementary material for: On the relationship of perceived inequality and policy preferences
Source: PLoS One. 2026 Aug 19;21(8):e0341298. doi: 10.1371/journal.pone.0341298 (PMC13489463; doi:10.1371/journal.pone.0341298)
Supplement: Supplementary file 19 [file pone.0341298.s019.pdf]

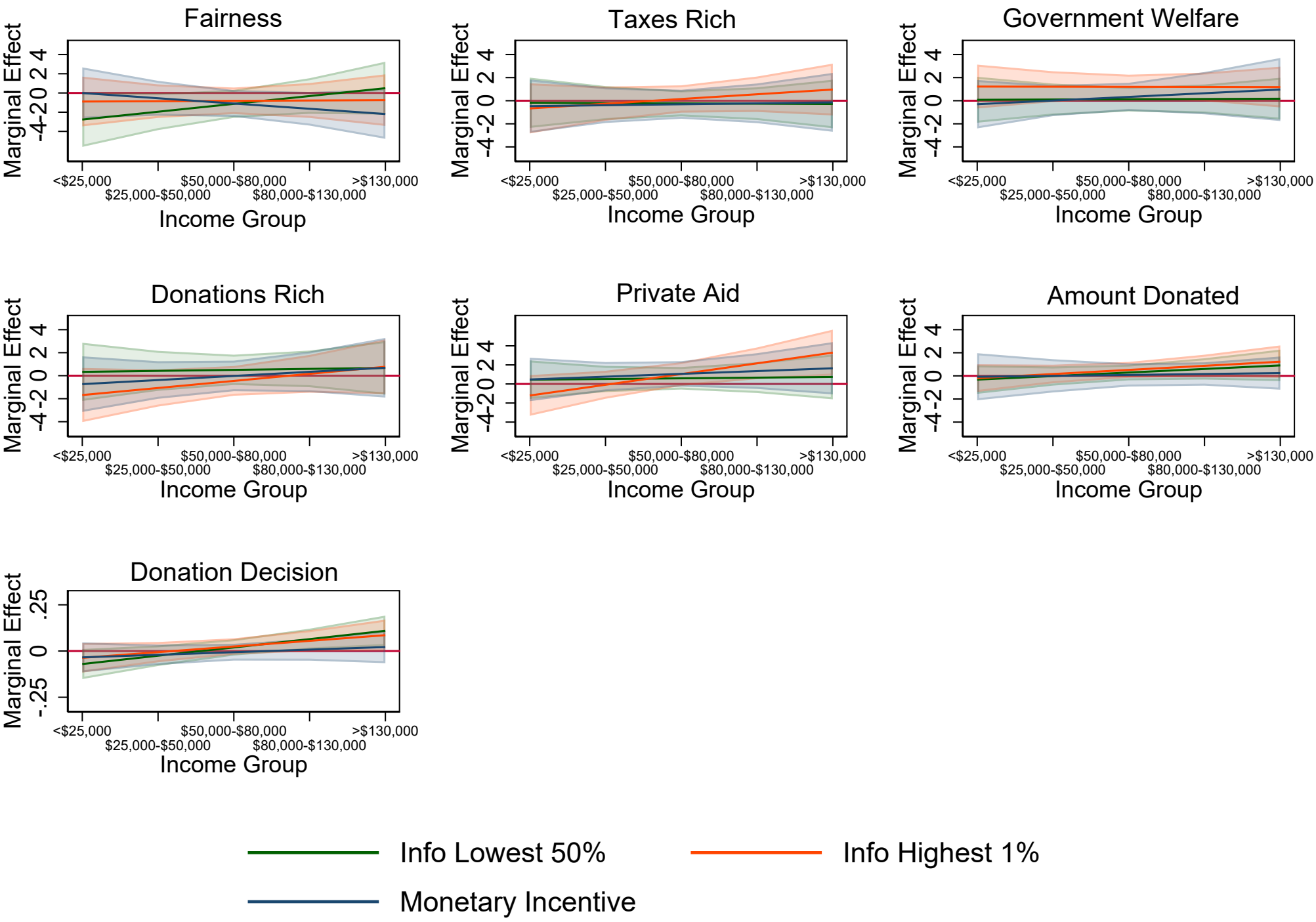

Fairness
Taxes Rich
Government Welfare
Donations Rich
Private Aid
Amount Donated
Donation Decision
Marginal Effect
Income Group
<$25,000
$25,000-$50,000
$50,000-$80,000
$80,000-$130,000
>$130,000
Info Lowest 50%
Info Highest 1%
Monetary Incentive
