# Supplementary material for: On the relationship of perceived inequality and policy preferences
Source: PLoS One. 2026 Aug 19;21(8):e0341298. doi: 10.1371/journal.pone.0341298 (PMC13489463; doi:10.1371/journal.pone.0341298)
Supplement: Supplementary file 22 [file pone.0341298.s022.pdf]

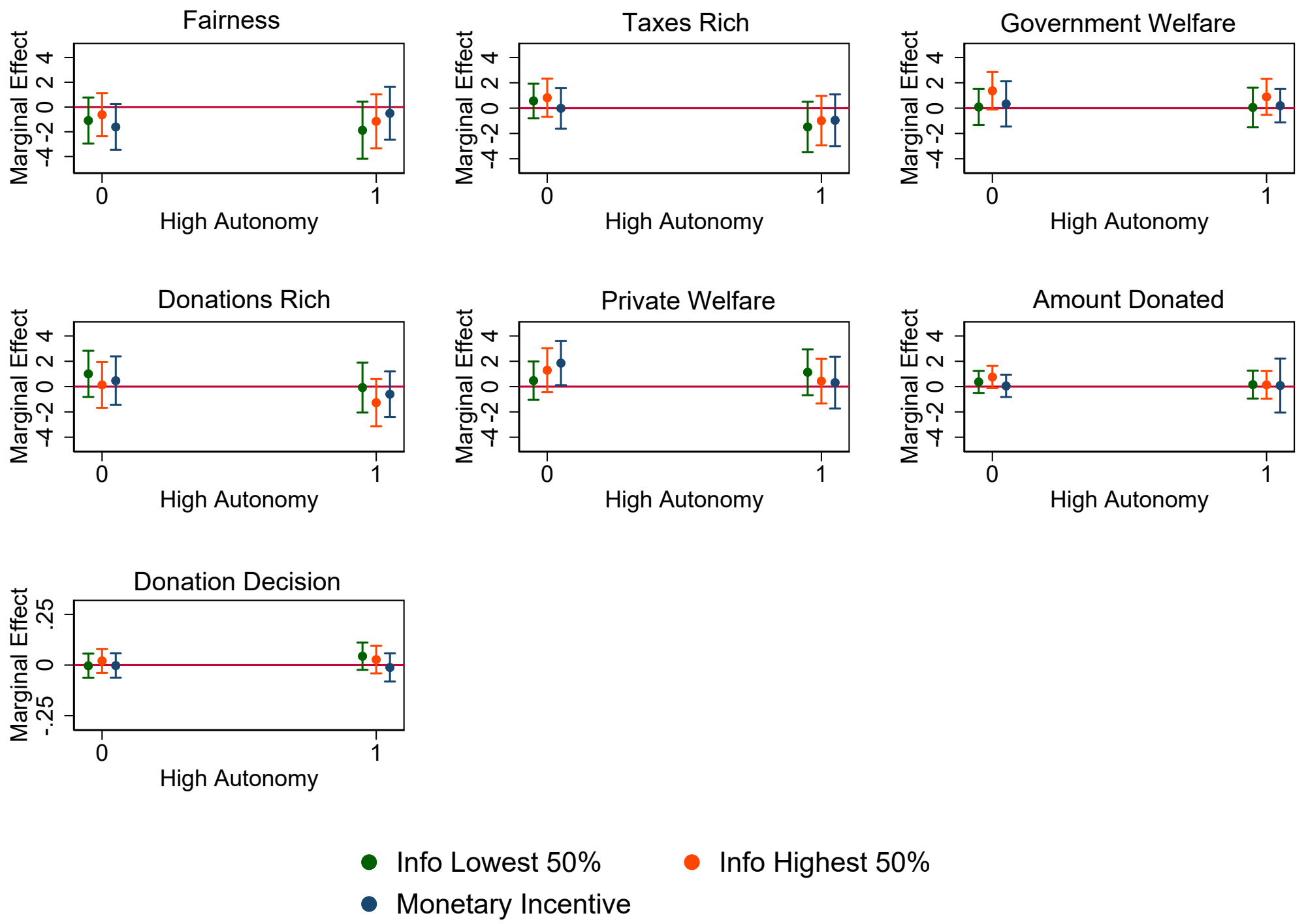
Fairness
Taxes Rich
Government Welfare
Donations Rich
Private Welfare
Amount Donated
Donation Decision
Marginal Effect
-4 -2 0 2 4
-.25 0 .25
High Autonomy
0
1
Info Lowest 50%
Info Highest 50%
Monetary Incentive
